# A Cross Modal Performance-Based Measure of Sensory Stimuli Intricacy

**DOI:** 10.1371/journal.pone.0147449

**Published:** 2016-02-03

**Authors:** Kobi Snitz, Anat Arzi, Merav Jacobson, Lavi Secundo, Kineret Weissler, Adi Yablonka

**Affiliations:** Dept of Neurobiology, Weizmann Institute of Science, Rehovot, Israel; University of Western Australia, AUSTRALIA

## Abstract

We define a new measure of sensory stimuli which has the following properties: It is cross modal, performance based, robust, and well defined. We interpret this measure as the intricacy or complexity of the stimuli, yet its validity is independent of its interpretation. We tested the validity and cross modality of our measure using three olfactory and one visual experiment. In order to test the link between our measure and cognitive performance we also conducted an additional visual experiment. We found that our measure is correlated with the results of the well-established Rapid Serial Visual Presentation masking experiment. Specifically, ranking stimuli according to our measure was correlated at r = 0.75 (*p* < 0.002) with masking effectiveness. Thus, our novel measure of sensory stimuli provides a new quantitative tool for the study of sensory processing.

## Introduction

In our everyday life we are surrounded by an enormous number of sensory stimuli. One of the brain’s most important functions is to integrate information from these stimuli, and to build representations of the world. In order to study how the brain represents sensory information, reliable and robust measures of sensory stimuli are needed. Two desirable features of stimulus measures which could make them a basis for systemic study of sensory perception are cross-modality and being based on performance.

A good cross-modal measure should consist of common properties which apply to stimuli across senses however most sensory stimulus measures known today are highly modality-specific and typically have little applicability to different modalities. After all one cannot smell the color red or see the smell of coffee. One well-known example for a cross modal measure is pleasantness; indeed, one can assess the pleasantness of all sensory stimuli.

Another desirable feature for a sensory stimulus measure is that it would be based on performance. In the context of psychophysics (and olfaction in particular) we refer to Wise et al. [1]: “Techniques based upon performance, rather than on the more common reporting of mental content, offer firmer possibilities for growth”. Under the definition of Wise et al. measures which are *derived* from assessments can still be performance based measures. For example, measuring the response times of assessments is a performance based measure.

For a more abstract example of derivation, consider the rating of odorants across a set of verbal descriptors (e.g. lemony, flowery, masculine, etc.). The first principal component (PC1) [2] of the ratings of all descriptors is well correlated with the ratings of pleasantness [3]. Hence PC1 can serve as a measure of pleasantness which does not depend on subjects’ assessment. We will show below that our measure, like PC1, is a derived measure which does not depend on the subjects assessments. i.e. it is a performance based measure.

We set out to define and test a novel cross modal measure of sensory stimuli and to demonstrate its applicability, consistency and cross modality. The measure we developed is based on the amount of variance in answers to any set of questions about the stimuli.

Furthermore, we propose an interpretation of our measure as a measure of the intricacy (or complexity) of the stimuli. We sketch an interpretation of our measure, but stress that its validity as a measure is independent of its interpretation.

Our interpretation is motivated by the intuition that more intricate stimuli will evoke a larger variance in the responses of test subjects. For example, the rating of a painting of a red square by Mark Rothko will be more consistent across subjects than the ratings of a Jackson Pollock painting. Accordingly, in the rest of this work we will provisionally refer to our measure as intricacy or variance measure.

Our interest in a measurement of intricacy is inspired by the hypothesis of Marr [4] of an algorithmic level of processing. The definition of intricacy is the sort of building block that might be used to carry out such analysis, in analogy to computational complexity, which underlies algorithmic analysis of machines.

We tested our measure with three separate olfactory experiments and a vision experiment. First, we demonstrated that the intricacy measure is consistent and independent of the descriptors used. Second, we showed that the intricacy measure is applicable across experiments and modalities. Finally, we showed that our intricacy measure is correlated with a measure of masking effectiveness in a rapid serial visual presentation (RSVP) experiment which is indicative of stimulus complexity.

## 1 Materials and Methods

The four initial experiments we conducted to define and validate our measure are listed here for reference throughout the paper.

**Experiment A** *Data set A consists of the ratings of 146 descriptors assigned to 10 odorants by 20 subjects (13 F). The descriptors were Hebrew translations of the ones used by A. Dravnieks in the “Atlas of Odor Character Profiles” [5].*

**Experiment B** *Data set B consists of ratings by 24 subjects (13 F) none of whom were involved in collecting the data in data set A. The subjects rated 10 odorants according to the 146 original (English) Dravnieks descriptors.*

Data set C contains ratings of the same odorants as in Data set B according to a different sets of descriptors. Unlike the ratings collected in the ‘Dravnieks’ atlas the descriptors used in data set C were of a different semantic type.

**Experiment C** *Data set C consists of ratings by 31 subjects (18 F) most of whom were involved in collecting the data in data set B. The subjects rated 10 odorants according to a list of 110 descriptors selected by K. Weissler [6]*

**Experiment D** *Data set D consists of ratings by 34 subjects (gender not recorded). The subjects rated 15 abstract visual textures according to a list of 51 descriptors. The descriptors included a mixture of those selected by K. Weissler. and Dravnieks descriptors. The stimuli textures were randomly chosen from the Brodatz texture set [7]*

### 1.1 Participants

A total of 109 subjects adults participated in 4 experiments: 3 independent olfactory experiments (data sets A B and C experiments A B C respectively) and one visual-cognitive experiment (data set D, experiment D). All subjects participated in the study after providing written informed consent to the procedures approved by the Ethics Committee at the Weizmann Institute of Science. None of the subjects reported on a history of olfactory dysfunction and all had a normal, or corrected to normal, vision.

All olfactory experiments were conducted in stainless steel-coated odorant-nonadherent rooms. All olfactory experiments used ∼ 40-s intertrial intervals, and trial order was counterbalanced across participants. Fresh odorant mixtures were prepared every 2 days. 20 subjects participated in experiment A (13 F), 24 subjects participated in experiment B (13 F), none of whom participated in experiment A, 31 (18 F) subjects participated in experiment C, and 34 (gender not recorded) subjects participated in experiment D.

### 1.2 Olfaction experiments

Subjects were invited to our lab and rated various olfactory stimuli along a range of verbal descriptors. In all olfactory experiments 10 odorants were presented to subjects in sniff-jars. The usage of the odorants in each experiment are detailed in S1 and S2 Tables. In addition S1 Fig shows the distribution of the selected odorants in a space of physicochemical descriptors.

Odorant ratings: Each subject rated the odorants along 146 verbal descriptors in experiment A, and B, and 110 verbal descriptors in experiment C (see list of descriptors in (S1 Text)). In experiment A test subjects used a 1-5 scale (as in [5]) to record their answers and in experiments B, C and D they used a visual analogue scales (VAS) on a cale of 1-100. For example, the question “to what extent does this odor smell like coconut” appeared above a computerized VAS scale ranging from “not at all” at one end, to “very much so” at the other. After sniffing the odorant presented in a jar, participants moved a computerized cursor to a point reflecting their perception. Odor and descriptors order were randomized, and inter-odor-interval was about 40 seconds. The descriptors in experiments A and B were Hebrew translations and English original descriptors respectively of the ones used by A. Dravnieks in the “Atlas of Odor Character Profiles” [5] while the descriptors in experiments C were the 110 descriptors selected by K. Weissler [6].

Data sets B, C and D were all collected by a custom built web application designed for collecting experimental data. [8]

In data sets B and C all but 4 and 5 subjects respectively repeated their ratings on different days. Our analysis was carried out on the averaged subject ratings when available and on the single ratings in other cases.

### 1.3 Vision experiment

Each subject rated the same 15 images which were selected at random from the Brodatz texture set [7] along 51 verbal descriptors using visual analogue scales (VAS). See list of descriptors in the supplementary materials (S2 Text) and see 3 for the images used in the experiment. The descriptors used for the Brodatz texture set images rating were identical to the descriptors used for odorant rating in experiment B. Images and descriptors orders were random across participants, and inter-image-interval was about 40 seconds.

The low level features of the images’ luminosity and spatial frequencies were analyzed using the same method (and code) used in [9]. In particular we calculated the luminosity of each image and the spatial frequencies along two directions. The spatial frequencies on a log scale in each direction were binned in 3 bins giving a total of 6 spatial frequency parameters. In addition we calculated a simple RMS contrast which is the root mean error square of the normalized image intensity values.

### 1.4 Rapid Serial Visual Presentation (RSVP) masking experiment

In this experiment each subject participated in one experimental session which consisted of 300 RSVP trials contained in 20 blocks of 15 trials. In each trial a randomly selected letter was flashed on the screen for 20-60 ms, followed by a masking image which was presented for 500 ms. The letters were selected from the 26 letters of the alphabet with the condition that no letter be presented twice within a block. The masking images were the same Brodatz textures used in experiment D presented in random order. All images were displayed using a custom built Matlab script which used the Psychtoolbox-3 extension [10][11]. In each trial the subject was required to indicate which letter was flashed before each masking image. After every block the performance of the subject was assessed. If the subject was correct on more than half of the trials in each block, the display duration of the letter was shortened by 10 ms. The initial display duration of the letters was 60 ms and the lower limit was set at 20 ms. After completion of all 300 trials, the percent of correctly identified letters for each masking image was calculated for each subject and then averaged across all individuals. The results is a measure of the effectiveness of each image at masking the letters. These values were later correlated with our measure of masking images’ intricacy.

## 2 Analysis

We propose to define the intricacy of a stimulus as the amount of variance in ratings of it according to different descriptors. The more consistent across different descriptors the amount of variance is, the more evidence there is for the validity of the definition. To test whether our definition is in fact consistent we use data from three different olfaction experiments and one vision experiment. See experiments A B C and D.

### 2.1 Preprocessing in two stages

An examination of the raw data revealed that the variance between subjects is smaller than the variance between odorants and between descriptors. Using the raw data, the variance between subject ratings is dominated by the other sources of variance and becomes undetectable. Therefore, as a preprocessing step, each subject’s data was first normalized using z-score across descriptors and then normalized again using z-score across stimuli. This was done since subjects might tend to give higher ratings according to one descriptor or odor than another. For reference we reiterate the two stages of preprocessing.

**Stage one** Subjects might tend to give higher ratings to one descriptor than another. We eliminated the variance due to differences between descriptors by taking z-scores across them. I.e. for each subject and stimulus we have a vector of ratings according to the different descriptors. We replace these ratings by the z-score calculated across the descriptors.

**Stage two** Subjects might tend to give higher ratings to one stimulus than another. We eliminate the variance due to differences between stimuli by taking z-scores across them. This stage of the data processing is applied to the data which has been normalized in stage one. For each subject and descriptor we have a vector of ratings (normalized as above) for each different stimulus. We replace these ratings with the z-score calculated across the stimuli.

Once these two sources of variance were normalized it was possible to observe the variance associated with a stimulus and a descriptor. That is, the variance for a given property and a given stimulus was calculated from the twice-normalized data across the different subjects (see more details in the following section). For each stimulus we then have a collection of variance values associated with the different descriptors. The higher these variances are the more *intricate* we define the stimulus to be.

### 2.2 Measuring the variance

To give numerical values and test the consistency of the variance property we need two definitions. It might be easier to understand the definitions of the stimulus and descriptor variance vectors as the columns and rows respectively of the following variance matrix.

$$V\;=\;\begin{array}{cc} & \begin{array}{cccc} o_{1} & o_{2} & \cdots & o_{n} \end{array}\\ \begin{array}{c} d_{1}\\ d_{2}\\ \vdots \\ d_{m} \end{array} & \left(\begin{array}{cccc} v_{11} & v_{12} & \cdots & v_{1n}\\ v_{21} & v_{22} & \cdots & v_{2n}\\ \vdots & \vdots & \ddots & \vdots \\ v_{m1} & v_{m2} & \cdots & v_{mn} \end{array}\right) \end{array}$$ (1)

The entry *v*_*ij*_ in the variance matrix is the variance in answers about stimulus *j* according to descriptor *i* calculated across subjects.

**Definition 1 (stimulus variance vector)** *Given an ordered list of n stimuli* {*o_1_…o_n_*} *and given a descriptor d (a.k.a property) of the stimuli, the stimulus variance vector of the descriptor is the vector*

$$\begin{array}{c} v_{d}=\left(v_{d,1},v_{d,2},\ldots v_{d,n}\right) \end{array}$$

*where v_d,i_ is the variance in the ratings (normalized as above) of property d for stimulus i calculated across test subjects.*

**Definition 2 (descriptor variance vector)** *Given a stimulus o and an ordered list of m descriptors denoted by* {*d_1_, d_2_, …, d_m_*} *the descriptor variance vector is the vector*

$$\begin{array}{c} v_{o}=\left(v_{1,o},v_{2,o},\ldots v_{m,o}\right) \end{array}$$

*where v_i,o_ is the variance in the ratings (normalized as above) of property i for stimulus o calculated across test subjects.*

The descriptor variance vector will be used in section 3.6 to distinguish between two stimuli, and in this section to define the intricacy of the stimulus.

**Definition 3 (intricacy of a stimulus)** *Given a stimulus o and an ordered list of m descriptors denoted by* {*d_1_,d_2_, …, d_m_*} *the intricacy of the stimulus o is the average of the descriptor variance vector of o with respect to the descriptors* {*d_1_,d_2_, …, d_m_*}

$$\begin{array}{c} I\left(o\right)=\frac{v_{1,o}+v_{2,o},\ldots +v_{m,o}}{m} \end{array}$$

*Where v_i,o_ is the variance in the ratings of property i for stimulus o calculated as above.*

In other words the intricacy of a stimulus is the average of the column corresponding to it in the matrix *V* in Eq 1.

## 3 Results

### 3.1 Our measure is robust and therefore well defined

Notice that our definition of intricacy (Definition 3) includes a choice of descriptors by which to calculate the intricacy. Our claim that the intricacy is a property of the stimulus amounts to saying that it is independent of the choice of descriptors. To what extent then is our claim that our measure is independent of the choice of descriptors justified ? In other words how different would the measure of intricacy be if we selected a different set of descriptors to measure it by ?

**Fig 1:**
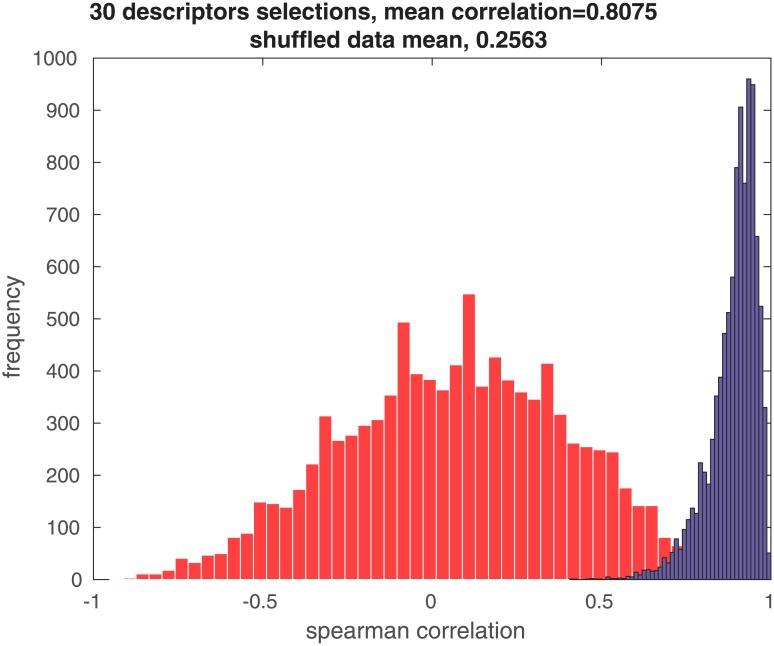
Rank Order correlation between intricacy ordering of the stimuli according to 10000 randomly selected pairs of 30 descriptors from data set A; Blue histogram is actual data with mean correlation of 0.8075 and red histogram is shuffled data with mean correlation of r = 0.2563; A two-sample Kolmogorov-Smirnov test comparing the two distributions has p value *p* < 10^−100^ that the distributions are equal.

To test this we selected two disjoint sets of 30 descriptors out of the 146 descriptors of data set A. If certain stimuli tend to have higher variance than others regardless of the descriptors then the intricacy of stimuli measured according to the two sets of descriptors would have the same relative size. In other words, the variances of the stimuli will retain their order according to mean variance regardless of the descriptors used to measure it. We calculated the intricacy of each of the 10 odorants according to the different sets of descriptors and calculated the rank order correlation between the variance measures determined by the two sets of descriptors. A histogram of 10000 such comparisons is shown in Fig 1. As can be seen in the figure, the mean correlation between measurements with different sets of descriptors is 0.8075. To asses the probability of such a collection of correlations we repeated the calculation on shuffled data (where the rows of the variance matrix were shuffled) and displayed the additional histogram in Fig 1. The probability of our result was measured with a Kolmogorov –Smirnov test between the two distributions of correlations and we get *p* < 10^−100^. This indicates that our measure is very robust and consistent under different choices of descriptors.

### 3.2 The dependence of intricacy on the number of the descriptors

**Fig 2:**
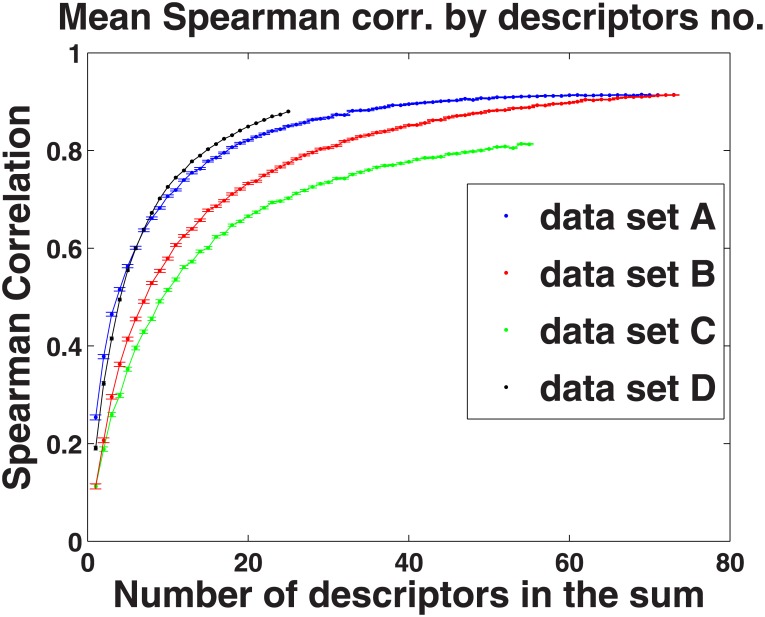
Number of odor variance vectors in a sum vs. average correlation between the sums: data sets A (blue), B (red), C(green), D(black).

One could fix a set of descriptors to serve as a fixed measure of intricacy; but given the high level of correlation between different choices of 30 descriptors, this is not necessary. One might want to trade off smaller amounts of data for a less stable measure for different applications. To test how the number of descriptors used to calculate stimulus intricacy would influence the measurement reproducibility we varied the number of descriptors used to generate our measure of intricacy, starting from 1 descriptor to 73, in data set A. For each number of descriptors we randomly selected 5000 non-overlapping pairs of sets of descriptors, and for each such pair we calculated the Spearman correlation between the intricacy according to the sets of descriptors. We found that the correlation is increased as the number of descriptors increases and it reaches about 90% of its maximum value at 30 descriptors. The trade-off reflecting the effect of the number of descriptors on the correlation level is shown in Fig 2 (blue line.)

We repeated this analysis for data sets B C and D and the results are also displayed in Fig 2. One can see that for all data sets, with sufficient descriptors the mean correlation between different sets of descriptors is greater than 0.7.

We also tested the limiting case of correlation between ranking according to single descriptors. For data sets A, B, C and D we calculated all possible comparisons between single descriptor ordering and plotted the histograms of Spearman correlations In S2 Fig. For data set A there are 146 × 145/2 comparisons between variance vectors and their correlations are shown in the histogram S2(a) Fig. We repeated the same analysis and found that the mean correlation between intricacy measures of all pairs of descriptors of experiment B and C was 0.14 and 0.11 respectively and significantly larger than the distributions of the shuffled data, with all p values *p* < 10^−43^ see the histograms in S2(B) and S2(C) Fig. This result shows that even one descriptor is enough to significantly inform on stimuli intricacy. Moreover as few as 30 descriptors are sufficient to reach a very high level of robustness.

### 3.3 Gender differences are inconclusive

For data sets A, B, C we have gender information and we tested the possibility that female and male test subjects give rise to a different ordering of the intricacies of the stimuli. In data set A, the correlation between the intricacy ordering according to females and males was r = 0.5758 p = 0.0878. We used bootstrapping to asses the statistical significance of this result, in particular we simulated 10000 splits of the test subjects and compared the correlations between ratings according to the different groups. The result of the simulation was that 95.41% of the splits had higher correlation. That is, the split of the data into groups of males and females produces ratings which are less alike than 95.41% of similar splits of the test subjects. We conclude that there is a significant difference between the intricacy measured from male subjects and that measured from the answers of female subjects. For data set B, the correlation between the intricacy ordering according to females and males was r = 0.7091 p = 0.0275, As above, when compared to 10000 simulated splits, 44.43% had higher correlation. This means that there is not enough evidence to conclude that men and women rate intricacy differently in this data set. For data set C, the correlation between the intricacy ordering according to females and males was r = 0.1394, p = 0.7072. Once more, when compared to 10000 simulated splits, only 6.89% had higher correlation. This means that there is not enough evidence to conclude that men and women rate intricacy differently in this data set.

Since only one of the three data sets for which we have gender information showed a just-significant difference between the intricacy calculated according to the male or female data; while the other two data sets showed differences which were far from significant; we can not conclude that there is a difference between groups of male and females in this measurement.

### 3.4 Our intricacy measure is applicable across modalities

**Fig 3:**
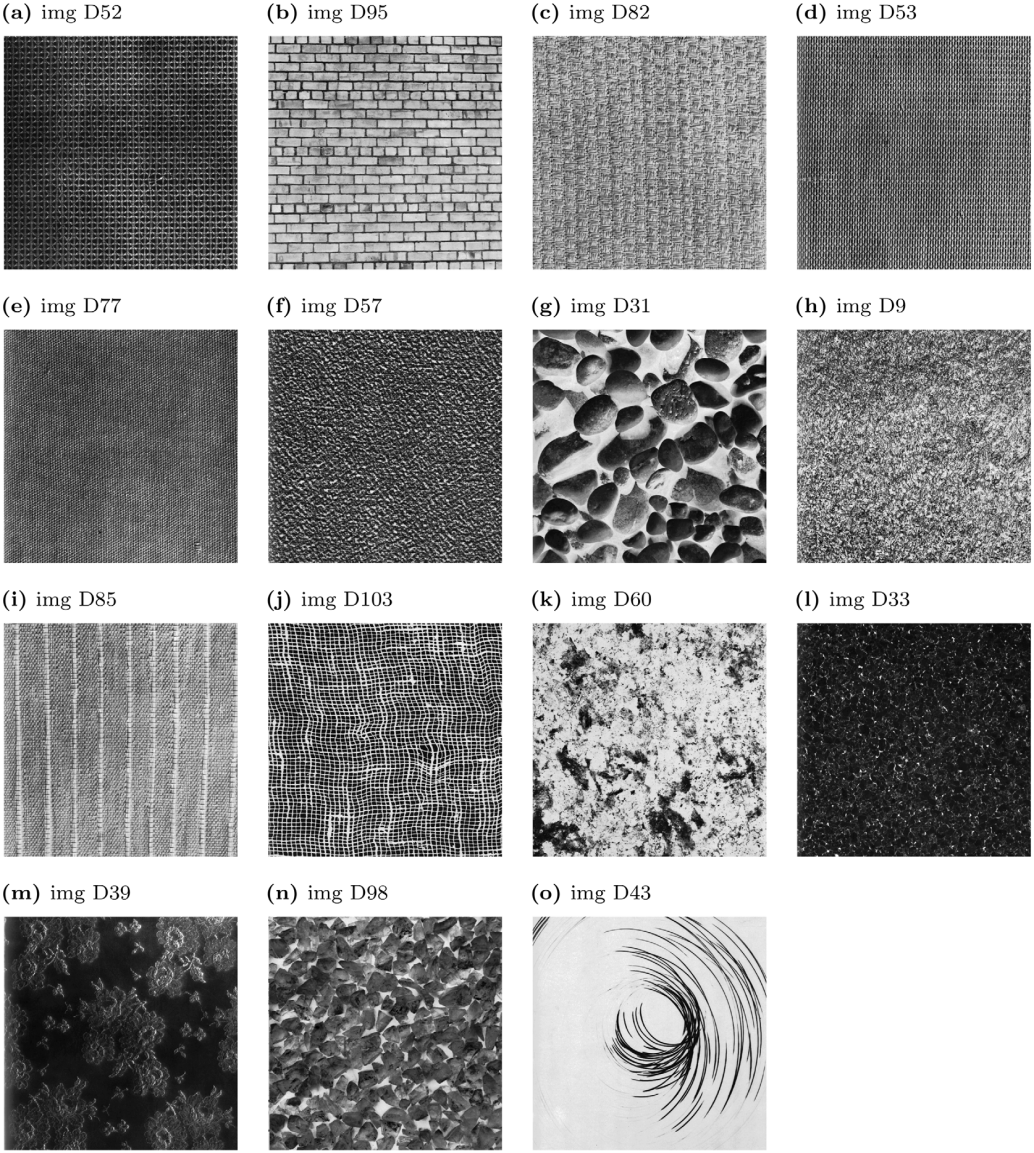
Brodatz figures listed in order of intricacy.

So far we showed that our measure of intricacy is applicable in olfactory experiments, however we still needed to test whether it is also applicable across sensory modalities. To test this we conducted a visual experiment where participants ranked 15 images of the Brodatz texture set (Fig 3) along 51 verbal descriptors (see list of descriptors in Supplementary materials S2 Text). In particular, since part of the purpose of the vision experiment was to test the applicability of the intricacy measure used in the olfactory data, the 51 descriptors used here were selected from the descriptors used in experiments A and B. Similarly to the results in the olfactory experiment we found that a ranking of stimuli intricacy constructed using a single descriptor is correlated with an average r = 0.189 and as above significantly larger than the shuffled data *p* < 10^−43^. However when the number of descriptors increases to 25 the correlation reaches 0.9. Thus, our measure of intricacy is modality independent and can be implemented both in olfaction and vision.

### 3.5 RSVP experiment shows intricacy to be a measure of perceptual load

In order for our definition of intricacy to be a useful tool one would also like to show that it is connected to other measurements of mental processing. To find such a property we conducted an additional vision experiment. We set out to find if there is a connection between the masking effectiveness of images and their intricacy. To that end we used a Rapid Serial Visual Presentation (RSVP) paradigm [12] with a set of Brodatz images [7] serving as masking images; see 3. To assess the masking effectiveness we calculated for each image the average percent of correctly identified letters proceeding it (see S3 Fig for the letter images used) We correlated the calculated image intricacy with the masking effectiveness and found that they are correlated at a Spearman correlation coefficient of r = 0.75, p = 0.0012.

As a check for confounding variables we further investigated whether or not our measure is related to standard low level image features. We found that neither intricacy nor masking effectiveness were significantly correlated with image luminance (r = 0.03 p = 0.89; r = 0.2 p = 0.47 respectively.) We also found that neither intricacy nor masking effectiveness were significantly correlated with image RMS contrast (r = -0.03 p = 0.92; r = 0.44 p = 0.11 respectively.) Of the 6 spatial frequency parameters 2 were significantly correlated with masking effectiveness (p = 0.0213 p = 0.0275 for low frequency bins at orientation 0 and *π*/2 respectively) and none of the 6 features were significantly correlated with intricacy. With a Bonferroni correction, none of the low level features are significantly correlated with either masking effectiveness or intricacy. Lastly, we used a single statistic to capture the frequency properties of the images by calculating the ratio of low frequencies (in both directions) to high frequencies (in both direction) for each image. This statistic was significantly correlated to masking effectiveness (r = 0.65 p = 0.01) but not significantly correlated to intricacy (r = 0.45 p = 0.09). We conclude that there is no significant evidence that our intricacy measure is mediated by this low-level image property.

This connection between intricacy and masking effectiveness suggests that there is a link between our measure of stimulus intricacy and stimulus processing demands. Previously it was suggested that higher stimulus intricacy will be manifested in higher perceptual load [13]. Thus, our measure of stimulus intricacy can be potentially used as an alternative consistent measure of perceptual load.

### 3.6 Distinguishing between stimuli

**Fig 4:**
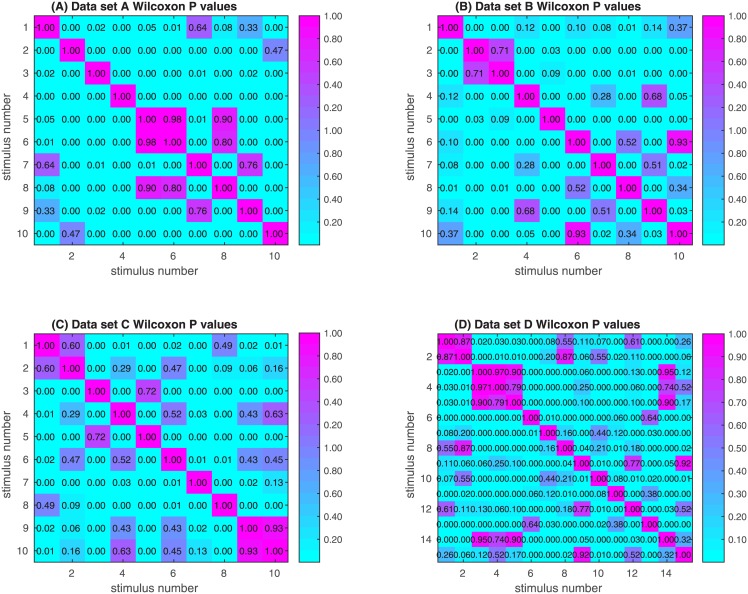
Data sets A, B, C and D heat maps of Wilcoxon correlation between descriptor variance vectors.

We showed that a set of stimuli can be rank-ordered by their intricacy and that this ranking is robust under change of descriptors. However this ranking does not enable one to measure the probability that the intricacy of one stimulus is distinguishable from the intricacy of another stimulus. To directly test whether two stimuli have significantly different intricacy we compared the distributions of the descriptor variance vectors values between all stimulus pairs. Since those distributions need not be normal and since the test is paired, we used a signed rank Wilcoxon test. We found that out of 45 stimuli pairs 37, 32, and 31 (in experiment A, B, C respectively) pairs had significant intricacy difference. (Fig 4(A), 4(B) and 4(C)) and in data set D 64 out of 105) (see Fig 4(D)). This result shows that not only is our measures of intricacy capable of rank-ordering stimuli but it can also significantly distinguish between different stimuli.

It should be noted that the similarity or even equality of two descriptor variance vectors does not undermine the validity of our measure. Just as two stimuli can have the same charge or mass they can also have the same intricacy. As mentioned above, the real test of our measure is its stability and independence of the choice of descriptors.

### 3.7 Familiarity pleasantness complexity and intricacy

Having defined a new measure of stimuli, it is natural to compare it to other common measures or measures of special relevance. We compared the measure of intricacy of the stimuli to ratings of “familiarity”, “pleasantness”, and “complexity”. See section 4.1 for discussion of these results.

Data sets C and D included as one of the descriptors a question about familiarity and we compared the average familiarity ratings of the odors and the relative amount of variance, see S2 and S3 Tables. The familiarity ratings and variance ratings were correlated at a Spearman correlation of r = 0.0909 p = 0.8114 for data set C and r = -0.1534 p = 0.5844 for data set D. We conclude that based on available evidence from the two data sets, there is no significant connection between rated familiarity and the amount of variance associated with a stimulus.

The only data set which contained ratings of pleasantness (rather than related descriptors) is data set C. We calculated the Spearman correlation of average pleasantness ratings and variance mounts of the odorants in data set C. We found that there was no significant correlation (r = 0.09, p = 0.8114). A second check of the connection between pleasantness and intricacy is to see if similar pleasantness ratings correlate with odorants which do not have significantly different intricacy distributions, see section 3.6. We calculated the correlation between the probabilities that odorants have the same variance distributions (the p-values in Fig 4) and the differences in average rated pleasantness, and obtained a correlation of r = 0.07 p = 0.64. We conclude that the data does not support a significant connection between pleasantness and the probability of identical intricacy distributions.

The only one of our data sets which included ratings of complexity is data set C. Using the same tests as above: the correlation between average rated complexity and measured intricacy we obtained a correlation of r = 0.4303 p = 0.2180. We conclude that the data does not support the hypothesis that there is a connection between measured intricacy and rated complexity. As above, we also calculated the correlation between the probabilities of identical variance distribution and differences in complexity ratings. The results were r = 0.1888 p = 0.2142 and so we conclude that the data does not support a connection between the rated complexity and probabilities of identical variance distributions.

## 4 Discussion

We defined a new measure of sensory stimuli that is linked to stimulus intricacy, this novel measure was derived from subjects’ performance rather than from subjects’ assessment. We should note that olfactory stimulus complexity has been linked to odor perception by the work of the Bsnsafi group along quite different lines than our approach [14]. We showed that our new measure is very robust (i.e. mostly independent of descriptors used) and its robustness increases as the number of descriptors used increases. Moreover, this measure is not only cross modal, but can also be computed exactly in the same manner in different sensory modalities. Importantly we show that it is also correlated with performance in a cognitive masking task, hence it can be an independent measure of perceptual load.

The fact that our measurement is applicable across modalities means that in principle the intricacy of a certain object can be measured according to different modalities. For example, one might ask if the intricacy of the odor of a peach should be related to the intricacy of an image of a peach. Since the two measures related to different senses there is no a priori reason for the two to be the same. In fact, if they do turn out to be the same, it would suggest that the measurement is not capturing the effect of the olfactory and visual sensation, but rather the connection to a mental image of a peach.

This raises the question of whether rating of some stimuli might be governed not by the stimulus property, but rather by a prior knowledge of the stimulus. This is a point which we address now.

### 4.1 Top-down effects

Consider a stimulus consisting of a line sketch of a dog such as would appear in a coloring book. When rating this stimulus according to one of the descriptors in our questionnaires (such as “masculine”), the subject can be influenced mostly by his or her knowledge, feelings, and even attitudes towards dogs. In an effort to reduce this effect, we restricted our attention to abstract stimuli, both in olfaction and in vision. If the amount of variance in answers reflected the degree to which a stimulus was connected to a mental image, it would interfere with the measurement of a *sensory* phenomenon. Therefore we wanted to verify that our measure of intricacy is not governed by stimuli familiarity. We set out to test whether there is a correlation between the rated familiarity of stimuli and our obtained measure of intricacy. In the olfactory experiments this was less of a problem. It is rare that people are able to name an odor [3]. To recreate this condition in the visual experiment we selected our stimuli from a collection of abstract images (see Fig 3). However, since the questions which subjects were asked about the stimuli do involve top down influence one might wonder how much top down influence determines the answers. The question is more acute in the case of the visual experiment since subjects are more likely to be able to give names or guess the source of the abstract images. However, in our follow-up RSVP experiment the letter identification task did not involve semantics. The fact that the masking images were shown for 500 ms further reduces the possibility that top down effects influenced subject responses. The fact that the RSVP experiment correlated significantly with our intricacy ordering means that semantics and top down effects do not determine our measure, which must therefore be related to the stimuli themselves.

Lastly, the process from sensation to answering a complex question surely involves high level processing but it does not follow that the response does not represent the stimulus. In a sense, the intricacy information is unrelated to the content of the descriptor. Consider that even when a number of the descriptors are unrelated to one or more of the modalities (such as “bitter” applied to images and “blue” applied to odorants) the effect of consistent variation is still detectable.

In addition to the above discussion of familiarity, it is a natural question to ask if our measure is correlated with other typical sensory measures. In particular, since three of our datasets are olfactory, we considered the correlation of average pleasantness ratings with intricacy ratings. Similarly, we tested the connection of the subject rated property of “Complexity” with our measure of intricacy. As detailed in section 3.7, our data does not support a significant connection between any of these measures and the measure of intricacy.

### 4.2 Relation to other measurements and future directions

We have demonstrated the robustness of our measure and showed that it is a property of the stimulus. We have also shown that our measure is significantly correlated with an independent well established measure (RSVP). However, we have not presented a hypothesis or evidence which relates our measure to measures such as chemical properties, which can be measured without test subjects. As an analogy we suggest the example of the colors of stimuli, their definition became more precise and perhaps more meaningful when they were related to electromagnetic wavelengths. However, even before the knowledge of wavelengths, colors were assigned to stimuli and could be measured directly or indirectly by subject response. Likewise, our measure can be measured even though it cannot (yet) be related to subject-independent measures.

We have demonstrated the robustness of our measure under a deliberately difficult test of applying olfactory descriptors to visual stimuli. In future work one might optimize the set of descriptors per modality to obtain even stronger results.

We have mentioned above that our motivation for defining our measure is the algorithmic analysis proposed by the work of Marr [4]. We propose that our measure will be used in future work as a test to compare alternative hypotheses about mental processing. For example, a future experiment could test if the intricacy of odorant mixture is connected to the number of odorants in the mixture. If there is a connection it would suggest that humans process odor mixtures as a collection of separate odors. If on the other hand, the intricacy of mixtures is not connected to the number of components, it would suggest that odorant mixtures are perceived as a new single entity. Along these lines, one could think of testing the intricacy of combination of image elements and perhaps sounds to test alternative hypotheses about the processing of combinations of stimuli. Another olfactory experiment could check if the intricacy of a diverse set of odors is divided into two clusters. Such a result could indicate the “innateness” of some odors versus other odors which were learned [15].

If such experiments are carried out and produce results we believe that this will mean that our measure is the basis for a new type of analysis of stimuli which is particularly suited for the analysis of the type suggested by Marr.

## Supporting Information

S1 Table Data set A odorants. (PDF)

S2 Table Data set C (and B) odorants. The mean familiarity is calculated for the raw data (no normalizing) and the variance is calculated from the twice z-scored data as usual. The Spearman correlation between the variance and familiarity is r = 0.0909 p = 0.8114.
(PDF)

S1 Fig Data sets A, B, C scatter of points in PC1 PC2 coordinates. Data sets A (in red), B, and C (in green) marked within a collection of 1363 odorant molecules commonly used in olfaction and odor production. The graph is produced as in [16]. The points marked in the figure are Isoamyl acetate (S) Nonane (I) Ethyl valerate (O) 5-methyl-2-hexanone (F) Isopropylbenzene (Cumene)(D) 1-pentanol (C) 1,7-octadiene (Q) 2-heptanone (G) 4-methyl-3-penten-2-one (mesityl oxide) (L) 3-methyl-2-buten-1-ol (P) Dibutyl Amine (J) Rthyl Pyrazine: 2-Ethyl Pyrazine (R) Eucalyptol (B) Hexanol: 1-Hexanol (H) Methyl Anthranilate (K) Valeric Acid Pentatonic Acid (E) Tolualdehyde: ortho- Tolualdehyde (N) Valeric Acid: iso Valeric Acid (M) Vanillin (A).
(EPS)

S1 Text List of descriptors used in the collection of dataset C. (PDF)

S2 Text List of descriptors used in the collection of dataset D. (PDF)

S2 Fig Data sets A, B, C and D. Histograms of Spearman correlations between all pairs of stimulus variance vectors. Data sets A, B, C and D. Histograms of Spearman correlations between all pairs of stimulus variance vectors. In all data sets the real (blue) and shuffled distributions (red) of correlations were compared with a Kolmogorov –Smirnov test and the probabilities of the distributions being the same was *p* < 10^−40^ in all cases.
(EPS)

S3 Fig Letters used in masking experiment. (TIF)

S3 Table Data set D textures the mean familiarity is calculated for the raw data (no normalizing) and the variance is calculated from the twice z-scored data as usual. The Spearman correlation between the variance and familiarity is r = -0.1536 p = 0.5844.
(PDF)

S4 Table Data sets A B and C odorants description. Cas numbers, chemical name and odorant descriptors which are collected from thegoodscentscompany.com [17] and the Atlas of odor character profiles [5].
(PDF)
