# Supplementary material for: A Cross Modal Performance-Based Measure of Sensory Stimuli Intricacy
Source: PLoS One. 2016 Feb 3;11(2):e0147449. doi: 10.1371/journal.pone.0147449 (PMC4740424; doi:10.1371/journal.pone.0147449)
Supplement: S1 Table — (PDF) [file pone.0147449.s001.pdf]

A cross modal performance-based measure of sensory stimuli intricacy

Kobi Snitz[1*,◑], Anat Arzi[1,◑], Merav Jacobson[1,◑], Lavi Secundo[1,◑], Kineret Weissler[1,◑], Adi Yablonka[1,◑]

1 Dept of Neurobiology, Weizmann Institute of Science, Rehovot, Israel

◑These authors contributed equally to this work.
*** kobi.snitz@weizmann.ac.il**

0.1 S1 Table

Data set A odorants.

Name	CAS number	mean variance
Isoamyl acetate	123-92-2	0.8119
Nonane	111-84-2	0.5398
Ethyl valerate	539-82-2	0.9723
5-methyl-2-hexanone	110-12-3	1.3859
Isopropylbenzene (Cumene)	98-82-8	0.7061
1-pentanol	71-41-0	0.6954
1,7-octadiene	3710-30-3	0.8330
2-heptanone	110-43-0	0.6895
4-methyl-3-penten-2-one (mesityl oxide)	141-79-7	0.8428
3-methyl-2-buten-1-ol	556-82-1	0.5233
