# Supplementary material for: A Cross Modal Performance-Based Measure of Sensory Stimuli Intricacy
Source: PLoS One. 2016 Feb 3;11(2):e0147449. doi: 10.1371/journal.pone.0147449 (PMC4740424; doi:10.1371/journal.pone.0147449)
Supplement: S2 Table — The mean familiarity is calculated for the raw data (no normalizing) and the variance is calculated from the twice z-scored data as usual. The Spearman correlation between the variance and familiarity is r = 0.0909 p = 0.8114. (PDF) [file pone.0147449.s002.pdf]

A cross modal performance-based measure of sensory stimuli intricacy

Kobi Snitz[1*,◕], Anat Arzi[1,◕], Merav Jacobson[1,◕], Lavi Secundo[1,◕], Kineret Weissler[1,◕], Adi Yablonka[1,◕]

1 Dept of Neurobiology, Weizmann Institute of Science, Rehovot, Israel

◕These authors contributed equally to this work.
*** kobi.snitz@weizmann.ac.il**

0.1 S2 Table

Data set C (and B) odorants The mean familiarity is calculated for the raw data (no normalizing) and the variance is calculated from the twice z-scored data as usual. The Spearman correlation between the variance and familiarity is r=0.0909 p=0.8114.

Name	Dravnieks Number	ordered by increasing data set C variance	ordered by increasing familiarity	Data set B variance
Isoamyl acetate	9	7 (0.7229)	4 (36.91)	0.6986
Dibutyl Amine	49	6 (0.6974)	10 (53.051)	1.0739
Rthyl Pyrazine: 2-Ethyl Pyrazine	61	9 (0.8225)	6 (40.3966)	0.9042
Eucalyptol	63	4 (0.6670)	1 (23.25)	0.6262
Hexanol: 1-Hexanol	75	10 (0.8230)	8 (50.95)	0.9697
Methyl Anthranilate	96	2 (0.6716)	2 (33.69)	0.8975
Valeric Acid Pentatonic Acid	111	1 (0.6138)	7 (49.30)	0.5739
Tolualdehyde: ortho-Tolualdehyde	134	8 (0.7366)	5 (37.55)	0.7975
Valeric Acid: iso Valeric Acid	141	2 (0.6533)	9 (53.050)	0.6564
Vanillin	143	3 (0.6554)	3 (34.05)	0.7311
