# Supplementary material for: A Cross Modal Performance-Based Measure of Sensory Stimuli Intricacy
Source: PLoS One. 2016 Feb 3;11(2):e0147449. doi: 10.1371/journal.pone.0147449 (PMC4740424; doi:10.1371/journal.pone.0147449)
Supplement: S1 Text — (PDF) [file pone.0147449.s004.pdf]

A cross modal performance-based measure of sensory stimuli intricacy

Kobi Snitz[1*,◑], Anat Arzi[1,◑], Merav Jacobson[1,◑], Lavi Secundo[1,◑], Kineret Weissler[1,◑], Adi Yablonka[1,◑]

1 Dept of Neurobiology, Weizmann Institute of Science, Rehovot, Israel

◑These authors contributed equally to this work.
*** kobi.snitz@weizmann.ac.il**

0.1 S1 Text

List of descriptors used in the collection of dataset C 1 : like ; 2 : airy ; 3 : erotic ; 4 : prominent ; 5 : make happy ; 6 : induces tension ; 7 : repulsive ; 8 : diluted ; 9 : penetrating ; 10 : strong ; 11 : smooth ; 12 : sour ; 13 : natural ; 14 : dry ; 15 : dark color ; 16 : nauseating ; 17 : healthy ; 18 : disgusting ; 19 : depressive ; 20 : harmonious ; 21 : familiar ; 22 : textured ; 23 : neutralizes odors ; 24 : salty ; 25 : Velvet-like ; 26 : dangerous ; 27 : stinking ; 28 : moldy ; 29 : appetizing ; 30 : itchy ; 31 : frightening ; 32 : annoying ; 33 : concentrated ; 34 : sweet ; 35 : sticky ; 36 : clean ; 37 : delicate ; 38 : deep ; 39 : decongestant ; 40 : red ; 41 : brown ; 42 : purple ; 43 : clear ; 44 : low sound ; 45 : noisy ; 46 : thin ; 47 : associated to a sound ; 48 : fragrant ; 49 : medical ; 50 : burnt ; 51 : pink ; 52 : green ; 53 : powdery ; 54 : edible ; 55 : aromatic ; 56 : bright ; 57 : masculine ; 58 : physically relaxing ; 59 : repellent ; 60 : compressed ; 61 : smarting ; 62 : new ; 63 : week ; 64 : hot ; 65 : hot (spicy) ; 66 : good ; 67 : tasty ; 68 : fresh ; 69 : old ; 70 : heavy ; 71 : chemical ; 72 : induces sneezing ; 73 : enjoyable ; 74 : attractive ; 75 : recalls an association ; 76 : sickening ; 77 : complex ; 78 : artificial ; 79 : dirty ; 80 : intriguing ; 81 : mysterious ; 82 : cause cough ; 83 : smoky ; 84 : surprising ; 85 : bitter ; 86 : emotionally relaxing ; 87 : intense ; 88 : fresh ; 89 : volatile ; 90 : rare ; 91 : pleasant ; 92 : stay ; 93 : feminine ; 94 : synthetic ; 95 : round ; 96 : murky ; 97 : cheerful ; 98 : sad ; 99 : blue ; 100 : yellow ; 101 : irritating sound ; 102 : high pitched ; 103 : cold ; 104 : moist ; 105 : reminiscent of a color ; 106 : bad ; 107 : poisonous ; 108 : rotten ; 109 : flat ; 110 : aggressive
