# Supplementary material for: A Cross Modal Performance-Based Measure of Sensory Stimuli Intricacy
Source: PLoS One. 2016 Feb 3;11(2):e0147449. doi: 10.1371/journal.pone.0147449 (PMC4740424; doi:10.1371/journal.pone.0147449)
Supplement: S2 Text — (PDF) [file pone.0147449.s005.pdf]

A cross modal performance-based measure of sensory stimuli intricacy

Kobi Snitz[1*,◑], Anat Arzi[1,◑], Merav Jacobson[1,◑], Lavi Secundo[1,◑], Kineret Weissler[1,◑], Adi Yablonka[1,◑]

1 Dept of Neurobiology, Weizmann Institute of Science, Rehovot, Israel

◑These authors contributed equally to this work.
*** kobi.snitz@weizmann.ac.il**

0.1 S2 Text

List of descriptors used in the collection of dataset D 1 : Non directional ↔ Directional ; 2 : Artificial ↔ Natural ; 3 : Not at all sweet ↔ Very sweet ; 4 : Small ↔ Large ; 5 : Not at all sour ↔ Very sour ; 6 : Very hot ↔ Very cold ; 7 : Convergence ↔ Divergence ; 8 : Very mild ↔ Very intense ; 9 : Rough ↔ Smooth ; 10 : Irregular ↔ Regular ; 11 : Non symmetrical ↔ Symmetrical ; 12 : Very appealing ↔ Very disgusting ; 13 : Very unpleasant ↔ Very pleasant ; 14 : Low feature density ↔ High feature density ; 15 : Closed ↔ Open ; 16 : Non granular ↔ Granular ; 17 : Not at all masculine ↔ Very masculine ; 18 : Low structural complexity ↔ High structural complexity ; 19 : Not at all salty ↔ Very salty ; 20 : Very heavy ↔ Very light ; 21 : Very smooth ↔ Very textured ; 22 : Very clean ↔ Very dirty ; 23 : Very fresh ↔ Very stale ; 24 : Near ↔ Far ; 25 : Old (created a long time ago) ↔ New (created recently) ; 26 : Non oriented ↔ Locally oriented ; 27 : Not at all burnt ↔ Very burnt ; 28 : Completely non-volatile ↔ Highly volatile ; 29 : Very repulsive ↔ Very attractive ; 30 : Not at all smoked ↔ Very smoked ; 31 : Not at all bitter ↔ Very bitter ; 32 : Nose stuffing ↔ Nose opening ; 33 : Static ↔ Dynamic ; 34 : Few ↔ Many ; 35 : Not at all familiar ↔ Very familiar ; 36 : Non random ↔ Random ; 37 : Non- uniform ↔ Uniform ; 38 : Not at all medicinal ↔ Very medicinal ; 39 : Non repetitive ↔ repetitive ; 40 : A part of ↔ Whole ; 41 : Low contrast ↔ High contrast ; 42 : Coarse ↔ Fine ; 43 : Curved ↔ Angular ; 44 : Highly poisonous, toxic ↔ Highly edible ; 45 : Very sharp ↔ Very dull ; 46 : Not at all green ↔ Very green ; 47 : Very annoying ↔ Very soothing ; 48 : Not at all erotic ↔ Very erotic ; 49 : Causes physical tension ↔ Causes physical relaxation ; 50 : Not at all feminine ↔ Very feminine ; 51 : Very dry ↔ Very wet
