# Supplementary figures and images for: A Cross Modal Performance-Based Measure of Sensory Stimuli Intricacy
Source: PLoS One. 2016 Feb 3;11(2):e0147449. doi: 10.1371/journal.pone.0147449 (PMC4740424; doi:10.1371/journal.pone.0147449)

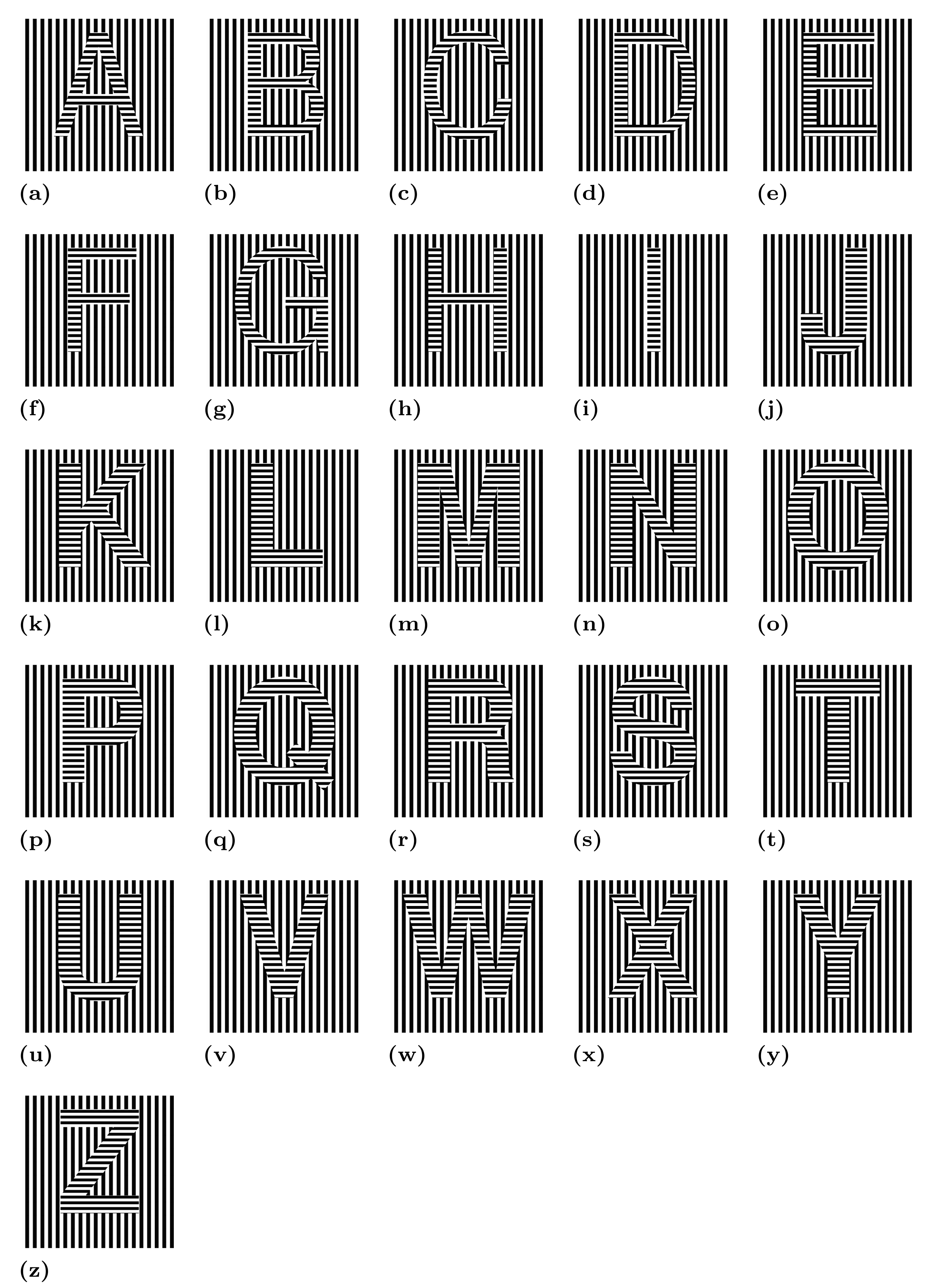

Supplement: S3 Fig — (TIF) [file pone.0147449.s007.tif]
