# Supplementary material for: A Cross Modal Performance-Based Measure of Sensory Stimuli Intricacy
Source: PLoS One. 2016 Feb 3;11(2):e0147449. doi: 10.1371/journal.pone.0147449 (PMC4740424; doi:10.1371/journal.pone.0147449)
Supplement: S3 Table — The Spearman correlation between the variance and familiarity is r = -0.1536 p = 0.5844. (PDF) [file pone.0147449.s008.pdf]

A cross modal performance-based measure of sensory stimuli intricacy

Kobi Snitz[1*,◑], Anat Arzi[1,◑], Merav Jacobson[1,◑], Lavi Secundo[1,◑], Kineret Weissler[1,◑], Adi Yablonka[1,◑]

1 Dept of Neurobiology, Weizmann Institute of Science, Rehovot, Israel

◑These authors contributed equally to this work.
*** kobi.snitz@weizmann.ac.il**

0.1 S3 Table

Data set D textures the mean familiarity is calculated for the raw data (no normalizing) and the variance is calculated from the twice z-scored data as usual. The Spearman correlation between the variance and familiarity is r=-0.1536 p=0.5844

Texture figure	variance	familiarity
(a)	0.5544	52.58
(b)	0.5789	84.20
(c)	0.6082	57.70
(d)	0.6641	48.52
(e)	0.6858	57.14
(f)	0.7167	46.50
(g)	0.7269	78.73
(h)	0.7632	44.20
(i)	0.7960	38.50
(j)	0.8120	55.97
(k)	0.8124	46.44
(l)	0.8796	47.29
(m)	0.8868	66.79
(n)	0.8891	64.32
(o)	0.9050	51.26
