# Supplementary material for: A Cross Modal Performance-Based Measure of Sensory Stimuli Intricacy
Source: PLoS One. 2016 Feb 3;11(2):e0147449. doi: 10.1371/journal.pone.0147449 (PMC4740424; doi:10.1371/journal.pone.0147449)
Supplement: S4 Table — Cas numbers, chemical name and odorant descriptors which are collected from thegoodscentscompany.com [17] and the Atlas of odor character profiles [5]. (PDF) [file pone.0147449.s009.pdf]

A cross modal performance-based measure of sensory stimuli intricacy

Kobi Snitz[1*,◑], Anat Arzi[1,◑], Merav Jacobson[1,◑], Lavi Secundo[1,◑], Kineret Weissler[1,◑], Adi Yablonka[1,◑]

1 Dept of Neurobiology, Weizmann Institute of Science, Rehovot, Israel

◑These authors contributed equally to this work.
*** kobi.snitz@weizmann.ac.il**

0.1 S4 Table

Data sets A B and C odorants description Cas numbers, chemical name and odorant descriptors which are collected from thegoodscentscompany.com [?] and the Atlas of odor character profiles [?].

Name	Dravnieks Number	Cas number	Good Scents descriptors	Dravnieks Descriptors
Isoamyl acetate	9	123-92-2	Sweet, banana, fruity with a ripe estry nuance Mosciano	Sweet Fruity (other than citrus) Banana Fragrant Aromatic
Nonane	NA	111-84-2	gasoline	NA
Ethyl valerate	NA	539-82-2	sweet fruity apple pineapple green tropical acidic apple berry	NA
5-methyl-2-hexanone	NA	110-12-3	NA	NA
Isopropylbenzene (Cumene)	NA	98-82-8	NA	NA
1-pentanol		71-41-0	fusel oil sweet balsam Pungent, fermented, bready, yeasty, winey solvent-like	NA
1,7-octadiene	NA	3710-30-3	NA	NA
2-heptanone	NA	110-43-0	fruity spicy sweet herbal coconut woody Cheese ketonic, green banana, with a creamy nuance	NA
4-methyl-3-penten-2-one (mesityl oxide)	NA	141-79-7	pungent earthy vegetable acrylic musty, mildew,chemical, card board like with nutty, chocolate and woody nuances	NA

3-methyl-2-buten-1-ol		556-82-1	fruity green lavender Sweet, fruity, alcoholic	
Dibutyl Amine	49	111-92-2	NA	Woody resinous Light Musty earthy Fragrant Aromatic
Rthyl Pyrazine: 2-Ethyl Pyrazine	61	13925-00-3	Nutty, musty, fermented, coffee, roasted,cocoa and meaty nuances woody peanut butter	'Peanut butter' 'Nutty' 'Woody resinous ' 'Musty earthy' 'Heavy'
Eucalyptol	63	470-82-6	eucalyptus herbal camphor	Camphor Medicinal Eucalyptus Cool cooling Turpentine (pine oil)
Hexanol: 1-Hexanol	75	111-27-3	ethereal fusel oil fruity alcoholic sweet green Pungent	Aromatic Fragrant Woody resinous Heavy Chemical
Methyl Anthranilate	96	134-20-3	fruity grape orange-flower neroli concord grape, musty with a floral powdery nuance	Burnt candle Fragrant Sweet Fruity other than citrus Aromatic
Valeric Acid Pentatonic Acid	111	109-52-4	sickening putrid acidic sweaty rancid Acidic and sharp cheese-like sour milky tobacco with fruity nuances	Sickening Putrid foul Rancid Sweaty Fecal (like manure)
Tolualdehyde: ortho-Tolualdehyde	134	529-20-4	cherry	Almond Aromatic Fragrant Sweet Cherry
Valeric Acid: iso Valeric Acid	141	503-74-2	sour stinky feet sweaty cheese tropical Cheese, dairy, acidic, sour, pungent, fruity, stinky, ripe fatty and fruity notes	Sickening Sweaty Rancid Fecal (like manure) Putrid foul
Vanillin	143	121-33-5	sweet vanilla creamy chocolate creamy and phenolic	Vanilla Sweet Chocolate Fragrant Aromatic
